# Honokiol Suppresses Glioma Cell Proliferation by Disrupting SUMO1-YAP1 Conjugation and Promoting YAP1 Proteasomal Degradation

**DOI:** 10.3390/biom16081158

**Published:** 2026-08-10

**Authors:** Zenghua Sheng, Qiaoyun Fang, Siqian Cui, Jian Wang, Huihui Xiao, Chunrong Wu, Debing Xiang

**Affiliations:** Department of Oncology, Jiangjin Hospital, School of Medicine, Chongqing University, Chongqing 402260, China; fqiaoyun0427@stu.cqu.edu.cn (Q.F.); cuisq742278794@cqu.edu.cn (S.C.); wj929@cqu.edu.cn (J.W.); 202337021069t@stu.cqu.edu.cn (H.X.); cqwcr6688@cqu.edu.cn (C.W.)

**Keywords:** GBM, honokiol, label-free proteomics, SUMOylation, ubiquitination, YAP1

## Abstract

Among primary central nervous system malignancies in adults, malignant glioma ranks first in incidence, with glioblastoma (GBM) standing as its most aggressive phenotype. This malignancy remains a devastating disease with limited therapeutic options, largely owing to the intricate network of dysregulated signaling pathways. SUMOylation, a post-translational modification that governs thousands of substrate proteins, is markedly hyperactivated in GBM and represents a rational but underexploited target. Here, we found that honokiol (HNK), a natural biphenolic compound extracted from the traditional Chinese medicine *Magnolia* species, suppressed SUMOylation in glioma cells, with a more prominent reduction in SUMO1-conjugated species than in SUMO2/3-conjugated species. Through a combination of label-free proteomic screening and functional biological assays, we pinpointed YAP1, a core Hippo pathway effector and established oncogenic driver in glioma cells, as a critical downstream target. Furthermore, our research indicated that inhibition of SUMOylation by HNK was associated with reduced levels of YAP1, resulting in glioma cell growth inhibition. Mechanistically, HNK induced YAP1 ubiquitin–proteasome degradation by disrupting SUMO1-YAP1 conjugation, leading to the subsequent blockade of YAP1 signaling. Collectively, our findings unveiled a previously unrecognized mechanism linking pharmacological deSUMOylation to YAP1 destabilization and emphasized the SUMO–YAP1 interface as a therapeutically actionable vulnerability. Overall, this work provides a strong rationale for developing HNK or its optimized derivatives as a first-in-class therapeutic strategy that addresses the network-level complexity of GBM.

## 1. Introduction

Gliomas, particularly glioblastoma (GBM), are among the most challenging brain tumors due to their clinically aggressive nature and dismal prognosis. Despite standard therapeutic interventions, including surgical resection followed by adjuvant chemoradiotherapy, the 5-year overall survival rate for affected patients remains below 5% [1,2,3]. Alarmingly, nearly all patients inevitably experience tumor recurrence, underscoring an urgent and unmet clinical need for innovative therapeutic strategies that move beyond conventional cytotoxic regimens and instead target the fundamental molecular drivers of glioma pathogenesis.

In recent years, the small ubiquitin-like modifier (SUMO) family, which includes four paralogs (SUMO-1, -2, -3, and -4) in mammalian cells, has garnered increasing attention as a critical regulator of oncogenic signaling [4,5,6,7]. SUMOylation, the dynamic post-translational conjugation of SUMO proteins to substrate lysine residues, governs a broad spectrum of essential cellular processes, including cell cycle progression, migration, and DNA damage repair [5,6]. To date, over 6000 proteins have been identified as SUMOylation targets, highlighting its pervasive influence on cellular homeostasis [7]. Importantly, aberrant hyper-SUMOylation is intimately linked to GBM biology. Compared with normal brain tissue, global SUMOylation levels are nearly 30-fold higher in GBM, and this elevation correlates closely with tumor development, progression, and therapeutic resistance [8,9]. Functional studies have demonstrated that disrupting SUMO-1-3 conjugation markedly suppresses DNA synthesis, clonogenic survival, and proliferation, while also sensitizing GBM cells to radiation [9]. Furthermore, our previous work revealed that SAE1, a core enzyme in the SUMOylation cascade, is significantly upregulated in both GBM clinical specimens and cell lines, where it promotes tumor growth and migration through enhancing Akt SUMOylation and phosphorylation [10]. Collectively, these findings position SUMOylation as a highly rational and promising therapeutic target for GBM intervention.

Among the potential pharmacological modulators, honokiol (HNK), a naturally occurring biphenolic compound with favorable blood–brain barrier permeability and low toxicity, has attracted considerable interest due to its multifaceted anti-cancer, anti-inflammatory, and anti-angiogenic activities [11,12,13,14,15,16,17,18]. For instance, HNK promotes death receptor-mediated apoptosis by downregulating c-FLIP in lung cancer and Nur77 in breast cancer [13,14]. Additionally, this compound has been shown to reverse resistance to both chemotherapy and radiotherapy across a broad spectrum of malignancies, including but not limited to oral squamous cell carcinoma, ovarian cancer, renal cell carcinoma, and head and neck squamous cell carcinoma [12,15,16,17,18]. In the GBM context, previous reports have indicated that HNK induces autophagy and apoptosis via reactive oxygen species-mediated p53 regulation and suppression of STAT3 signaling, respectively [19,20]. Despite these insights, however, the precise molecular mechanisms underlying the effects of HNK on GBM, particularly its potential interplay with protein SUMOylation, remain largely unexplored.

In the present study, we identified HNK as a novel SUMOylation inhibitor that effectively suppressed glioma cell growth and induced apoptosis. Our data indicated that HNK preferentially inhibited SUMO1 conjugation over SUMO2/3. Moreover, we provided evidence that the inhibition of SUMOylation by HNK was associated with reduced levels of YAP1, resulting in glioma cell growth inhibition. Mechanistically, HNK induced YAP1 ubiquitin–proteasome degradation by disrupting SUMO1-YAP1 conjugation, ultimately resulting in the inactivation of the YAP1 signaling pathway. Taken together, our findings not only uncovered a previously unrecognized mode of action for HNK but also emphasized the therapeutic potential of pharmacologically targeting the SUMO-YAP1 interface. This work thus proposes a novel perspective for exploiting aberrant SUMOylation as a viable strategy to combat glioma and offers new avenues for developing more effective therapies against this devastating disease.

## 2. Materials and Methods

### 2.1. Reagents and Antibodies

Cell culture media DMEM and fetal bovine serum (FBS) were ordered from Gibco in Waltham, MA, USA. MG132 (HY-13259), N-ethylmaleimide (NEM) (HY-D0843), and cycloheximide (CHX) (HY-12320) were ordered from MedChemExpress in New Jersey, NJ, USA. HNK (S2310) was obtained from Selleck Chemicals in Houston, TX, USA. Slurry anti-Flag M2 affinity gel (A2220) was ordered from Sigma-Aldrich in St. Louis, MO, USA. The protein-A beads (161-4013) were bought from Bio-Rad in Hercules, CA, USA.

All commercial antibodies used in this study were obtained from the following suppliers: Myc (HA600018), Flag (BX00086), His (BX00085-C3), SUMO1 (ET1606-53), SUMO2/3 (ET1701-17-100), SAE1 (JG35-88), SAE2 (ET1705-73), UBC9 (ET1610-21), Ubiquitin (ET1609-21), YAP1 (ET1608-30), p-YAP1 (Ser127) (ET1611-69-10), Bcl-2 (JF104-8), and Lamin B1 (R15508-1) were all ordered from HuaBio Company in China; LATS1 (9153S) and p-LATS1 (Thr1079) (8654S) were obtained from Cell Signaling Technology in Danvers, MA, USA; CYR61 (sc-271217) and CTGF (sc-365970) were obtained from Santa Cruz Biotechnology in Dallas, TX, USA; and goat anti-mouse Alexa Fluor 594 (A21044) and goat anti-rabbit Alexa Fluor 594 (A32740) were obtained from Invitrogen in Carlsbad, CA, USA. The antibody of β-tubulin (TA-10, Zsbio, Chongqing, China) was used to quantify the expression of housekeeping gene β-tubulin for comparison normalization. The IgG antibody (A7016, Beyotime, Shanghai, China) was taken as a nonspecific binding control for IP performance.

### 2.2. Cell Culture

HEK293T, H4, U138 and T98G cells were obtained from the American Type Culture Collection. U251 cells were purchased from the European Collection of Authenticated Cell Cultures. We also performed STR authentication for all cell lines to confirm their identities. All cell lines were cultured with DMEM, including 10% FBS and 100 units of penicillin-streptomycin in a 37 °C incubator with 5% CO_2_ and 95% air.

### 2.3. Detection of Cell Proliferation

The growth of HNK-treated cells was analyzed by CCK8 according to our previous methods [4,10]. Cells were plated in 96-well plates at 5000 cells per well and cultured overnight, then subjected to different treatments (0, 5, 10, 20, 40, 50, 55, 60, 70, 80, and 100 μM) for 24 h. The procedure has previously been described in detail [4,10]. For the colony formation assay, cells (3000 cells/well) were cultured in 6-well plates with different treatments for 10 days. The colonies were washed with PBS and fixed with 4% paraformaldehyde, then stained with Giemsa. The visible colonies were counted using ImageJ 1.42q software.

### 2.4. Cell Apoptosis

Cell apoptosis was detected using a double-staining apoptosis detection kit, following the protocol (KGA106, Nanjing KeyGen Biotech Co., Ltd., Nanjing, China). The procedure has previously been described in detail [10]. Briefly, the culture supernatants containing detached cells were saved, and the remaining adherent cells were gently trypsinized using 0.25% trypsin-EDTA (without phenol red) at 37 °C for 2–3 min, then pooled with the corresponding floating cells to ensure inclusion of all apoptotic and dead cells. After washing twice with ice-cold PBS, the combined cell pellets were resuspended in 500 μL of binding buffer, and 10 μL of Annexin V-FITC and 10 μL of propidium iodide (PI) were added. The mixtures were incubated for 15 min at room temperature in the dark. For each sample, at least 10,000 events within the live-cell gate were acquired. The gating strategy was as follows: debris and cell doublets were first excluded using a forward-scatter (FSC) versus side-scatter (SSC) dot plot; singlet cells were then gated based on FSC-H/FSC-W discrimination. Compensation controls were prepared using single-stained samples (Annexin V-FITC only and PI only) and an unstained control to set appropriate fluorescence compensation. Apoptotic populations were defined as early apoptotic (Annexin V-FITC^+^/PI^−^), late apoptotic (Annexin V-FITC^+^/PI^+^), and necrotic (Annexin V-FITC^−^/PI^+^) cells; viable cells were Annexin V-FITC^−^/PI^−^. Data analysis was performed using CytExpert v2.6 software.

### 2.5. Immunofluorescence

Cells were seeded onto the glass cover slips in 6-well plates (1 × 10^4^ cells/well), then treated with 60 μM HNK for 12 h. After being fixed with 4% paraformaldehyde in PBS for 30 min, cells were then washed three times with PBS and permeabilized with 0.4% Triton X-100 for 15 min, followed by blocking with 5% FBS for 1 h. The treated cells were incubated with indicated primary antibody overnight at 4 °C and subsequently incubated with Alexa Flour secondary antibody at 37 °C for 1 h. Nuclei were finally stained with DAPI for 10 min. Lastly, images were visualized using a Zeiss LSM 510 confocal microscope (Carl Zeiss AG, Oberkochen, Germany).

### 2.6. Plasmids and Cell Transfection

Plasmids pHis-SUMO1 and pMyc-SUMO1 were used and were stored in our laboratory [4]. The pHA-ubiquitin plasmid was provided by Prof. Hu H. [21], and the pFlag-YAP1 plasmid was provided by Huang C [22] (State Key Laboratory of Biotherapy, Sichuan University, Chengdu, China). Plasmids were transiently transfected into HEK293T, U251 and H4 cells with the transfection reagent (Lipofectamine2000, 11668–019, Life Technologies, Carlsbad, CA, USA) to observe biological effects.

### 2.7. Immunoprecipitation (IP) and Western Blot

Previous studies have shown that the activation of YAP1 depends on cellular density, where under low cell density conditions YAP localizes to the nucleus and modulates the transcription of genes involved in cell growth and survival [23]. Therefore, the number of cells in each group must be approximately the same before HNK treatment. In general, cells were seeded in triplicate and grown until 70% confluence. Protein lysates were prepared with the NP40 buffer containing protease inhibitor cocktail and 20 mM N-ethylmaleimide, and then quantified by BCA Protein Assay Kit (Thermo Fisher Scientific, Waltham, MA, USA, Catalog No.23250). Equal amounts of soluble proteins (15–30 μM) were used for Western blot analysis. For IP, the supernatants of lysates containing approximately 2 mg protein were incubated with 50 μL of slurry anti-Flag M2 affinity gel or protein-A beads overnight at 4 °C. Cell lysates or protein samples from co-IP were separated on a 7.5–12.5% SDS-PAGE gel to test the protein expression abundance against specific antibodies using Western blot. The corresponding secondary antibody was incubated with the PVDF membrane for 1 h at room temperature. Signal detection was detected by ECL (EMD Millipore, Burlington, MA, USA, WBKLS0500).

### 2.8. Quantitative RT-PCR

Total RNAs were extracted using Trizol reagent (Catalog NO.15596-026, Invitrogen, Carlsbad, CA, USA) according to the manufacturer’s instructions and used to synthesize complementary DNA utilizing HiScript II Q RT SuperMix (Nanjing Vazyme Biotech Co., Ltd., Nanjing, China). Then, quantitative real-time PCR (qRT-PCR) was performed using a Bio-Rad CFX96 system with the SYBR green SuperMix. The procedure has been described in detail previously [24].

The forward RT-PCR primer for YAP1 was 5′-GCTACAGTGTCCCTCGAACC-3′, and the reverse one was 5′-TCCTTCCAGTGTTCCAAGGT-3′. The forward one for β-actin was 5′-CACCCAGCACAATGAAGATC-3′, and its reverse was 5′-CTGATCCACATCTGCTGGAA-3′.

### 2.9. Liquid Chromatography with Tandem Mass Spectrometry (LC-MS/MS)

Label-free proteomic analysis was carried out of proteomic alterations upon 60 μM HNK treatment in U251 cells for 24 h. A total of 200 μg protein samples were processed according the FASP approach protocol [25,26]. Protein samples were digested by trypsin, and peptides were identified by liquid chromatography–tandem mass spectrometry (LC-MS/MS) on an easy nano-LC1000 HPLC system (Thermo Scientific, San Jose, CA, USA) and Q-Exactive mass spectrometry (Thermo Scientific, San Jose, CA, USA) [26]. For MS analysis, peaks in the mass range of *m*/*z* 300–1800 were used to generate a peptide mass fingerprint that was searched against in the human Swiss-Prot database (version 2019.12), using the MaxQuant search engine (version 2.2, Matrix Sciences, London, UK). The procedure has been described in detail previously [26].

### 2.10. Statistics Analysis

The significance of differences was determined using Student’s *t* test or two-way ANOVA. All quantitative data were expressed as means ± S.D. *p* < 0.05 was regarded as a significant difference.

## 3. Results

### 3.1. HNK Suppresses Glioma Cell Growth

**Figure 1:**
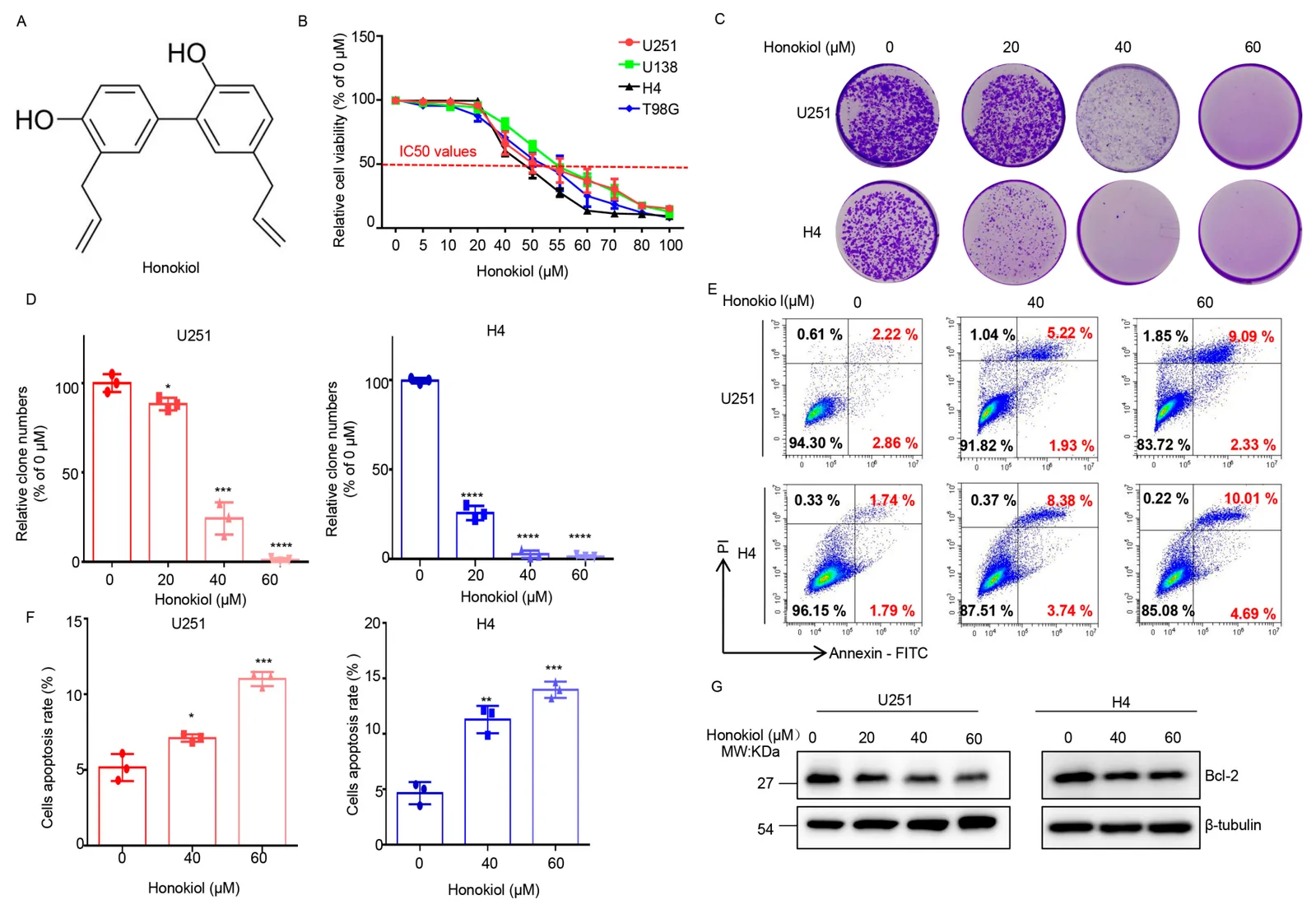
HNK inhibits cell proliferation and induces apoptosis of glioma cells. (**A**) The chemical structure of HNK. (**B**) The CCK8 assay of various glioma cell lines treated with indicated concentrations of HNK for 24 h. (**C**,**D**) The colony formation assay of U251 and H4 cells treated with indicated concentrations of HNK. Representative images (**C**) and quantification of clone numbers (**D**) are shown. (**E**,**F**) Flow cytometric analysis of apoptosis in U251 and H4 cells treated with indicated concentrations of HNK for 24 h. Representative images (**E**) and quantification of cell apoptosis rate (**F**) are shown. (**G**) Western blot analysis of Bcl-2 expression in U251 and H4 cells treated with indicated concentrations of HNK for 24 h. Data are means ± s.d. and are representative of 3 independent experiments. *, *p* < 0.05; **, *p* < 0.01; ***, *p* < 0.001; ****, *p* < 0.0001. The original Western blot images can be found in the Supplementary Materials.

To determine the effects of HNK on glioma cells, we assessed cell growth across a panel of glioma cell lines (U251, U138, T98G, and H4). CCK-8 assay results exhibited a dose-dependent inhibition of cell viability in all lines (Figure 1A), with IC_50_ values ranging from 40 to 60 µM (Figure 1B). Consistently, colony formation assays further showed that HNK significantly suppressed the proliferation of U251 and H4 cells (Figure 1C,D). To evaluate the pro-apoptotic effects, we performed Annexin V/PI double staining. Treatment with 60 µM HNK for 24 h induced a marked increase in apoptosis in both cell lines (Figure 1E,F). Moreover, HNK treatment reduced the expression of the anti-apoptotic protein Bcl-2 (Figure 1G). Collectively, these results indicate that HNK not only inhibits glioma cell proliferation but also promotes apoptosis.

### 3.2. HNK Inhibits SUMO1-Conjugation and SUMO1 Over-Expression Partially Attenuates HNK-Induced Inhibition of Proliferation in Glioma Cells

**Figure 2:**
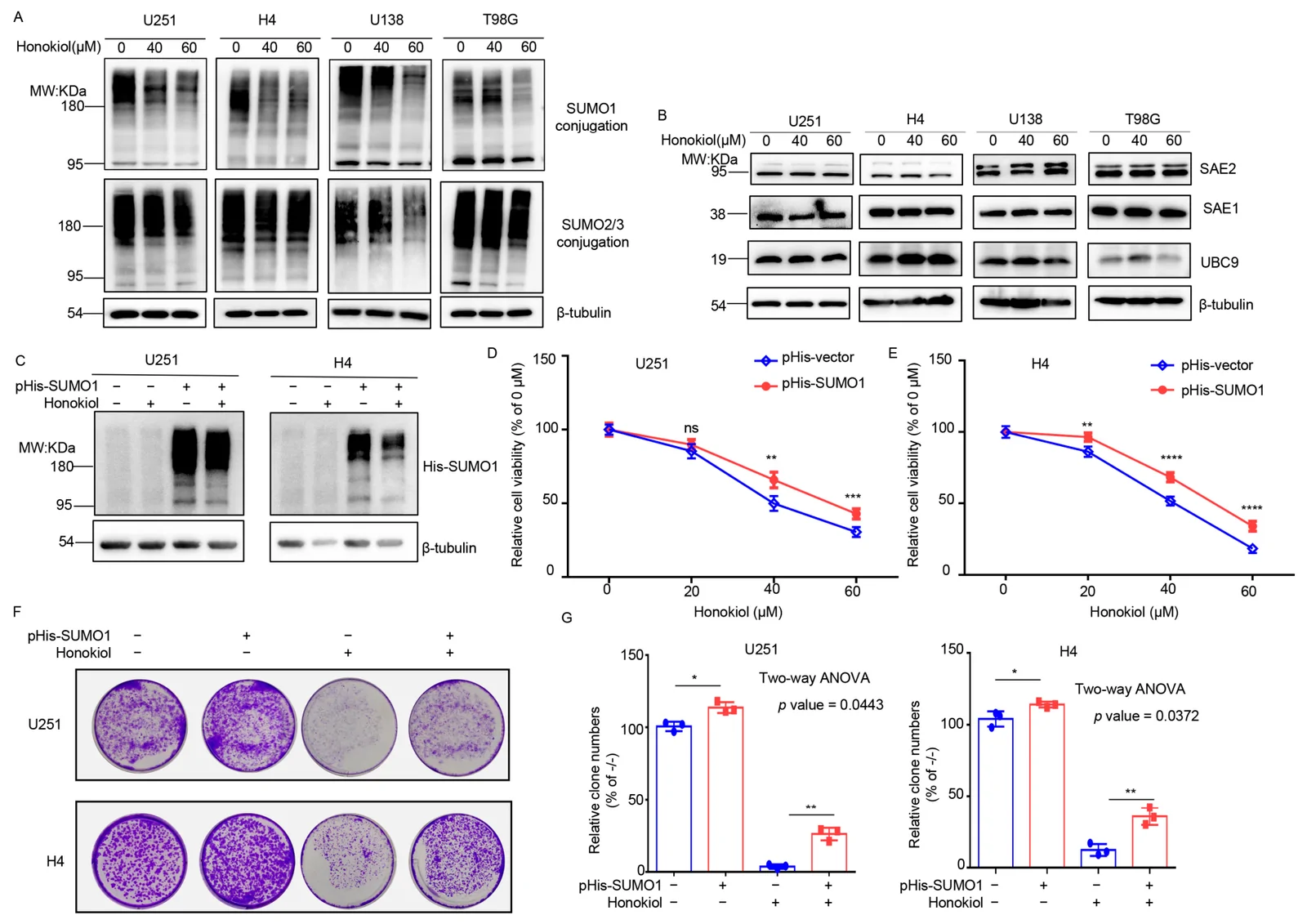
HNK suppresses cell viability and colony formation in glioma cells by decreasing global levels of SUMO1-conjugation. (**A**) Western blot analysis of global SUMO1-conjugation and SUMO2/3-conjugation expression in U251 and H4 cells treated with indicated concentrations of HNK for 24 h. High molecular weight (>95 kDa) SUMO-1 and SUMO-2/3 conjugates were cropped in each lane and the total intensities were measured. (**B**) Western blot analysis of the expression of SUMO signaling components (including SAE1, SAE2, and UBC9) in U251 and H4 cells treated with indicated concentrations of HNK for 24 h. (**C**) Western blotting analysis of His-SUMO1 expression conducted in His-SUMO1-transfected U251 and H4 cells treated with or without 60 μM HNK for 24 h. (**D**,**E**) Cell viability assessed by CCK-8 assay in His-SUMO1-transfected U251 (**D**) and H4 (**E**) cells following 24 h treatment with indicated concentrations of HNK. (**F**,**G**)The colony formation assay of His-SUMO1-transfected U251 and H4 cells treated without or with 40 μM HNK. Representative images (**F**) and quantification of clone numbers (**G**) are shown. Data were analyzed by ordinary two-way ANOVA, which indicted a significant interaction between SUMO1 over-expression and HNK treatment in U251 cells (*p* value = 0.0443) and H4 cells (*p* value = 0.0372). Data are means ± s.d. and are representative of 3 independent experiments. ns, not significant; *, *p* < 0.05; **, *p* < 0.01; ***, *p* < 0.001; ****, *p* < 0.0001. The original Western blot images can be found in the Supplementary Materials.

Previous evidence has indicated that certain bisphenol compounds and their derivatives can inhibit protein SUMOylation in vitro [27]. To ascertain whether HNK inhibits protein SUMOylation in glioma cells, we examined the levels of SUMO1 and SUMO2/3 conjugation. Four glioma cell lines were treated with 40 and 60 μM HNK for 24 h, based on our previous results showing that these concentrations effectively inhibited cell proliferation. We found that 60 μM HNK inhibited SUMO1 conjugation more pronouncedly than SUMO2 conjugation (Figure 2A). Moreover, we examined the effects of HNK on the cascade enzymes in the SUMO pathway, including SAE1, SAE2, and UBC9. Notably, HNK did not significantly alter the expression levels of these enzymes (Figure 2B). Thus, HNK was capable of significantly reducing global SUMO1 conjugation levels in glioma cells.

To further evaluate the role of SUMO1 in mediating the effects of HNK, U251 and H4 cells were transfected with the pHis-SUMO1 plasmid to over-express SUMO1. As shown in Figure 2C, Western blot analysis showed a pronounced increase in SUMO1 conjugation, indicating successful SUMO1 over-expression. Moreover, SUMO1 over-expression significantly attenuated the HNK-induced inhibition of cell viability (Figure 2D,E). Consistently, cell proliferative ability was assessed by colony formation assay. As expected, the inhibitory effect of 40 μM HNK was attenuated by SUMO1 over-expression (Figure 2F,G). Collectively, these findings indicate that SUMO1 plays a critical role in the cellular response to HNK and suggest that HNK functions as a SUMO inhibitor in suppressing glioma cell growth.

### 3.3. Label-Free LC-MS/MS Analysis of HNK-Induced Proteomic Alterations in Glioma Cells

**Figure 3:**
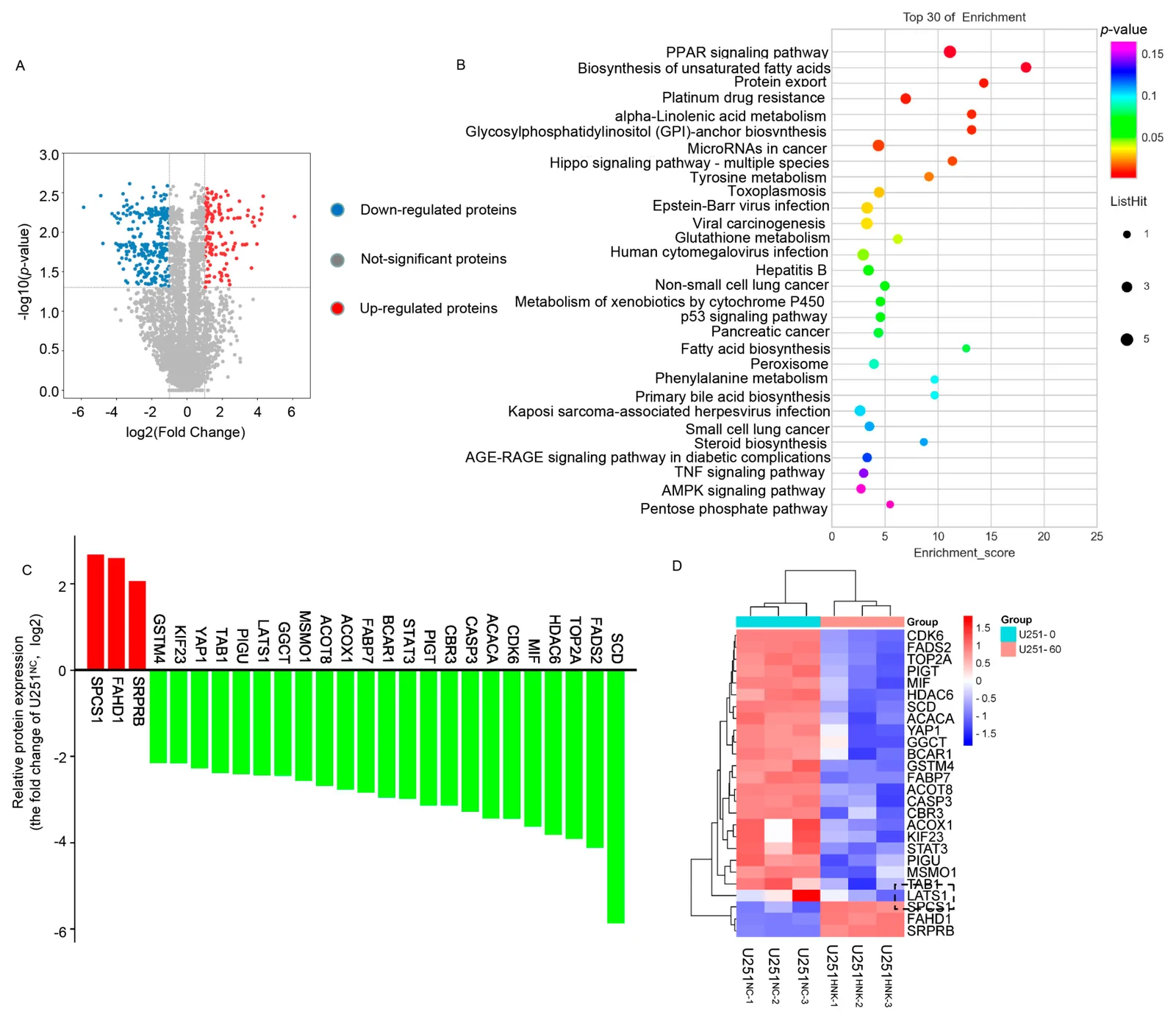
Label-free LC-MS/MS analysis of HNK-induced proteomic alterations in U251 cells. (**A**) Volcano plot showing differentially expressed proteins upon 60 μM HNK treatment in U251 cells for 24 h. The abscissa represents the log2 fold value of the fold difference in the expression of proteins in both groups, and the ordinate represents the negative logarithm of the *p* value of the changes in proteins. (**B**) DEPs which were analyzed by KEGG pathway enrichment were involved in the top 30 pathways. (**C**,**D**) HNK-mediated proteins expression changes in the top 30 pathways.

To further analyze global proteomic alterations resulting from HNK-mediated inhibition of protein SUMOylation, we compared the proteomic expression profiles between DMSO- and 60 μM HNK-treated U251 cells. Following HNK treatment, a total of 198 differentially expressed proteins (DEPs) were identified, exhibiting a greater than 2-fold change with a *p*-value < 0.05 (Figure 3A), as detailed in Supplementary Table S1. We next performed KEGG pathway enrichment analysis, which revealed that the statistically significant DEPs were enriched in various signaling pathways, including the Hippo pathway (Figure 3B, Supplementary Table S2). Among the significant DEPs, YAP1, a core component of the Hippo pathway, was markedly downregulated in HNK-treated U251 cells (Figure 3C,D). Collectively, these results suggest that HNK remodels the Hippo signalling pathway in U251 cells.

### 3.4. HNK Suppresses YAP1 Protein Expression, and YAP1 Over-Expression Partially Attenuates HNK-Induced Inhibition of Proliferation in Glioma Cells

**Figure 4:**
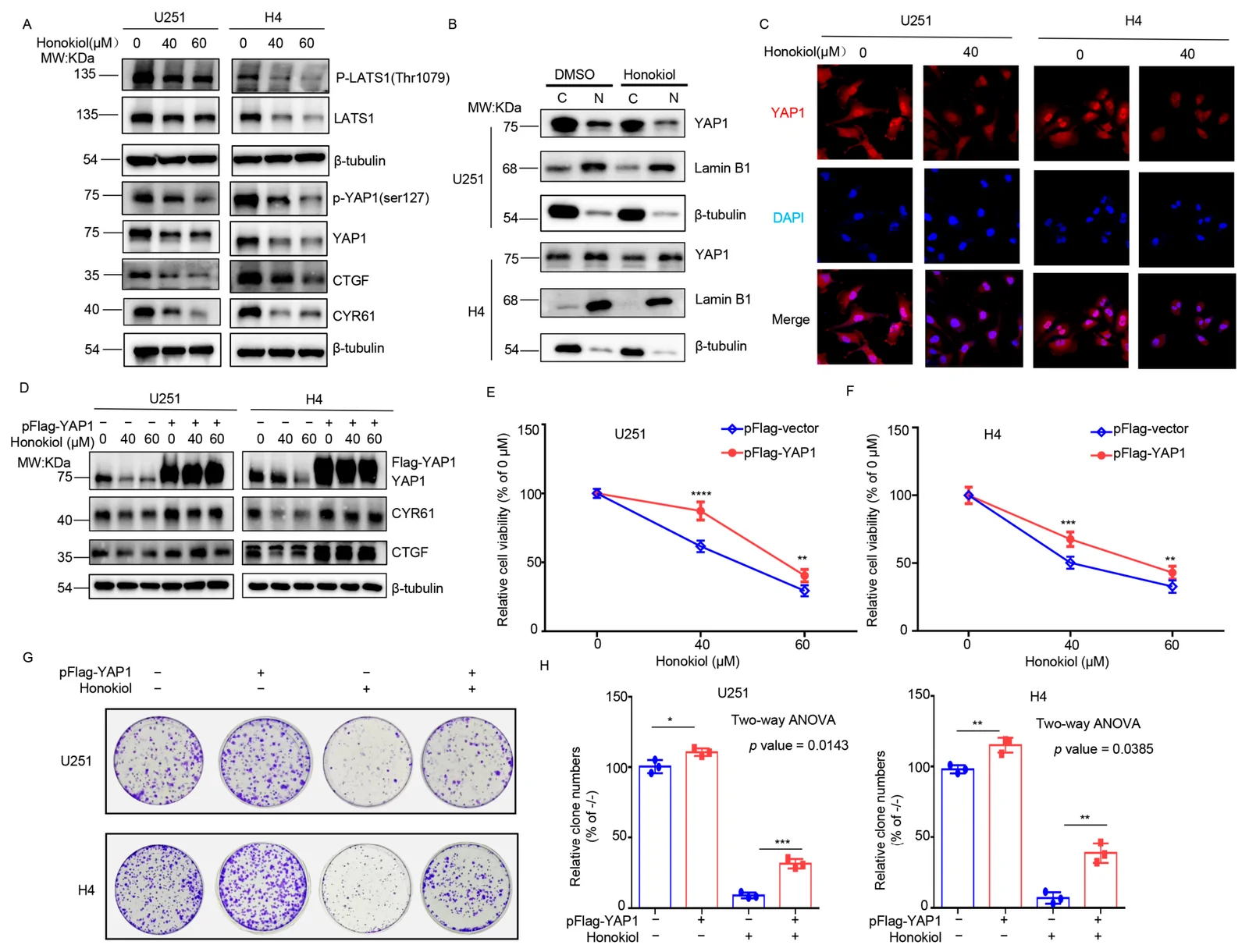
HNK suppresses YAP1 protein expression in glioma cells. (**A**) Western blot analysis of expression of Hippo signaling components (including p-LATS1, LATS1, p-YAP1, YAP1, CYR61 and CTGF) in U251 and H4 cells treated with indicated concentrations of HNK for 24 h. (**B**) The nuclear/cytosol fractionation assay and Western blot analysis show YAP1 translocation in U251 and H4 cells after 40 μM HNK treatment for 24 h. Lam B1 and Tubulin used as nucleus and cytoplasmic marks, respectively. (**C**) Immunofluorescence assay used to displaying the location of YAP1 in U251 and H4 cells after 40 μM HNK treatment for 24 h. (**D**) Western blotting analysis of the CYR61 and CTGF expression in YAP1-transfected U251 and H4 cells treated indicated concentrations of HNK for 24 h. (**E**,**F**) Cell viability assessed by CCK-8 assay in Flag-YAP1-transfected U251 (**E**) and H4 (**F**) cells following 24 h treatment with indicated concentrations of HNK. (**G**,**H**) The colony formation assay of Flag-YAP1-transfected U251 and H4 cells treated without or with 40 μM HNK. Representative images (**G**) and quantification of clone numbers (**F**) are shown. Data were analyzed by ordinary two-way ANOVA, which indicted a significant interaction between YAP1 over-expression and HNK treatment in U251 cells (*p* value = 0.0143) and H4 cells (*p* value = 0.0385). Data are means ± s.d. and are representative of 3 independent experiments. *, *p* < 0.05; **, *p* < 0.01; ***, *p* < 0.001; ****, *p* < 0.0001. The original Western blot images can be found in the Supplementary Materials.

Given that Hippo signaling plays a pivotal role in glioma progression and that YAP1 is a target protein of SUMO1 [28], we next examined whether HNK could regulate YAP1 protein expression. Consistent with the label-free proteomic analysis, HNK significantly suppressed YAP1 protein expression in glioma cells (Figure 4A). Of note, although the phosphorylation of LATS1 (a known inhibitory kinase of YAP1) was also inhibited, the YAP1 downstream targets CTGF and CYR61 were markedly decreased (Figure 4A). These data indicate that HNK primarily acts on the Hippo pathway via direct suppression of YAP1, rather than via LATS1-mediated regulation. Moreover, nuclear accumulation of YAP1 is essential for its transcriptional activity and oncogene activation [29]. Our data showed that HNK treatment reduced nuclear YAP1 expression compared with the DMSO control (Figure 4B). This was further corroborated by immunofluorescence staining, which revealed diminished nuclear YAP1 fluorescence (red) upon HNK treatment (Figure 4C). Collectively, these results indicate that HNK suppresses YAP1 protein expression in glioma cells.

To further evaluate the role of YAP1 in mediating the effects of HNK, U251 and H4 cells were transfected with the pFlag-YAP1 plasmid to over-express YAP1. As expected, Western blot analysis showed a significant increase in YAP1 expression (Figure 4D). Meanwhile, the expression levels of CYR61 and CTGF were elevated in HNK-treated glioma cells transfected with pFlag-YAP1 compared to control cells (Figure 4D). Moreover, the CCK-8 assay showed that YAP1 over-expression significantly attenuated the HNK-induced inhibition of cell viability (Figure 4E,F). Consistently, cell proliferative ability was also assessed by colony formation assay. As expected, the inhibitory effect of 40 μM HNK was attenuated by YAP1 over-expression (Figure 4G,H). Collectively, these findings suggest that HNK-induced YAP1 downregulation is critical for suppressing glioma cell proliferation.

### 3.5. HNK-Induced Promotion of YAP1 Ubiquitylation and Destabilization Is Partially Counteracted by SUMO1 Over-Expression

**Figure 5:**
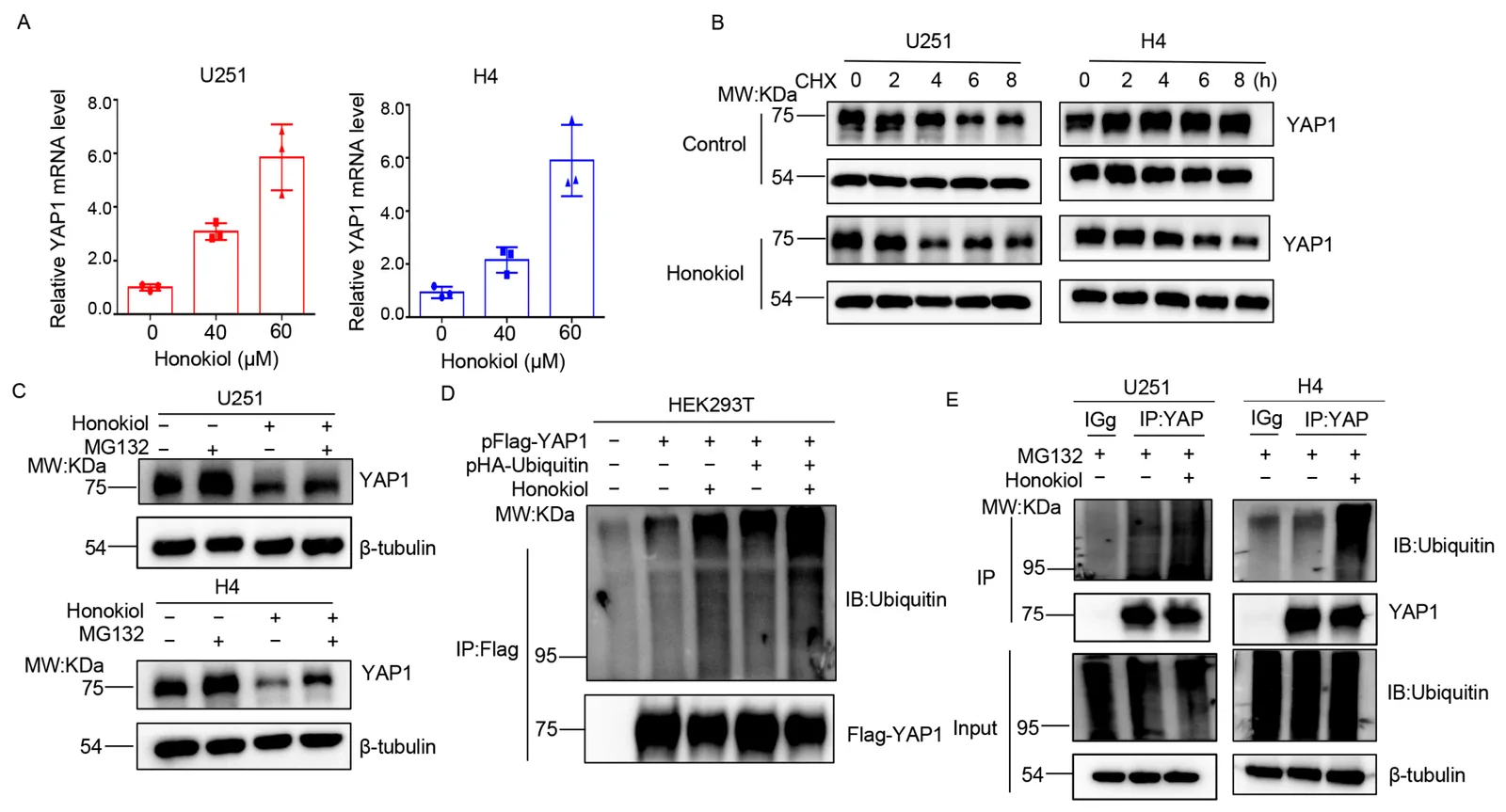
HNK downregulates YAP1 protein by triggering ubiquitination. (**A**) Q-PCR analysis of YAP1 in U251 and H4 cells treated with indicated concentrations of HNK for 24 h. Expression is normalized to Actin mRNA. Data are represented as the mean ± s.d. (**B**) To evaluate the effect of HNK on YAP1 stability, U251 and H4 cells were treated with 60 µM HNK in the presence or absence of 100 µM CHX for the indicated time kinetics. (**C**) U251 and H4 cells were treated with 60 µM HNK for 24 h, then treated with or without 20 μM MG132 for 8 h before harvest. Then, Western blotting analysis of the YAP1 expression in U251 and H4 cells was carried out. (**D**) To evaluate the effect of HNK on YAP1 ubiquitination, HEK293T cells were transiently co-transfected with pFlag-YAP1 and pHA-ubiquitin plasmids for 24 h, then treated with 60 μM HNK for 24 h. Cell lysates were performed with IP to capture Flag-tagging YAP1, which was immunoblotted with FLAG and ubiquitin antibodies. (**E**) To analyze the ubiquitination of endogenous YAP1 after 60 μM HNK treatment for 24 h, U251 and H4 cells were treated with 20 μM MG132 for 8 h before harvest. Cell lysates were performed with IP to capture YAP1, which was immunoblotted with YAP1 and ubiquitin antibodies. (Ub)n-Flag-YAP1: the ubiquitinylated Flag-tagging YAP1, (Ub)n-YAP1: the ubiquitinylated YAP1, IP: immunoprecipitation, IB: immunoblot, Input: same account of cell lysate to load. The original Western blot images can be found in the Supplementary Materials.

To further explore the mechanism underlying HNK-suppressed YAP1 expression, we examined YAP1 mRNA levels following HNK treatment. Unexpectedly, as shown in Figure 5A, HNK upregulated YAP1 mRNA, which suggested that the reduction in YAP1 protein is not attributable to transcriptional suppression but rather to post-translational regulation, specifically protein degradation. To test this possibility, we first assessed YAP1 protein stability in the presence of cycloheximide (CHX), a protein biosynthesis inhibitor. HNK accelerated YAP1 protein degradation compared with the control (Figure 5B). Given that the ubiquitin–proteasome system plays a critical role in YAP1 stability and degradation [28], we next examined whether HNK-induced YAP1 downregulation depends on proteasome activity using the proteasome inhibitor MG132. Indeed, MG132 partially reversed the HNK-mediated reduction in YAP1 (Figure 5C), which indicated that HNK decreases YAP1 expression through proteasomal degradation. We then analyzed HNK-induced YAP1 ubiquitination by IP with anti-Flag or anti-YAP1 antibodies. As shown in Figure 4D,E, HNK increased YAP1 ubiquitination. Collectively, these findings indicate that HNK promotes YAP1 degradation via the ubiquitin–proteasome system.

**Figure 6:**
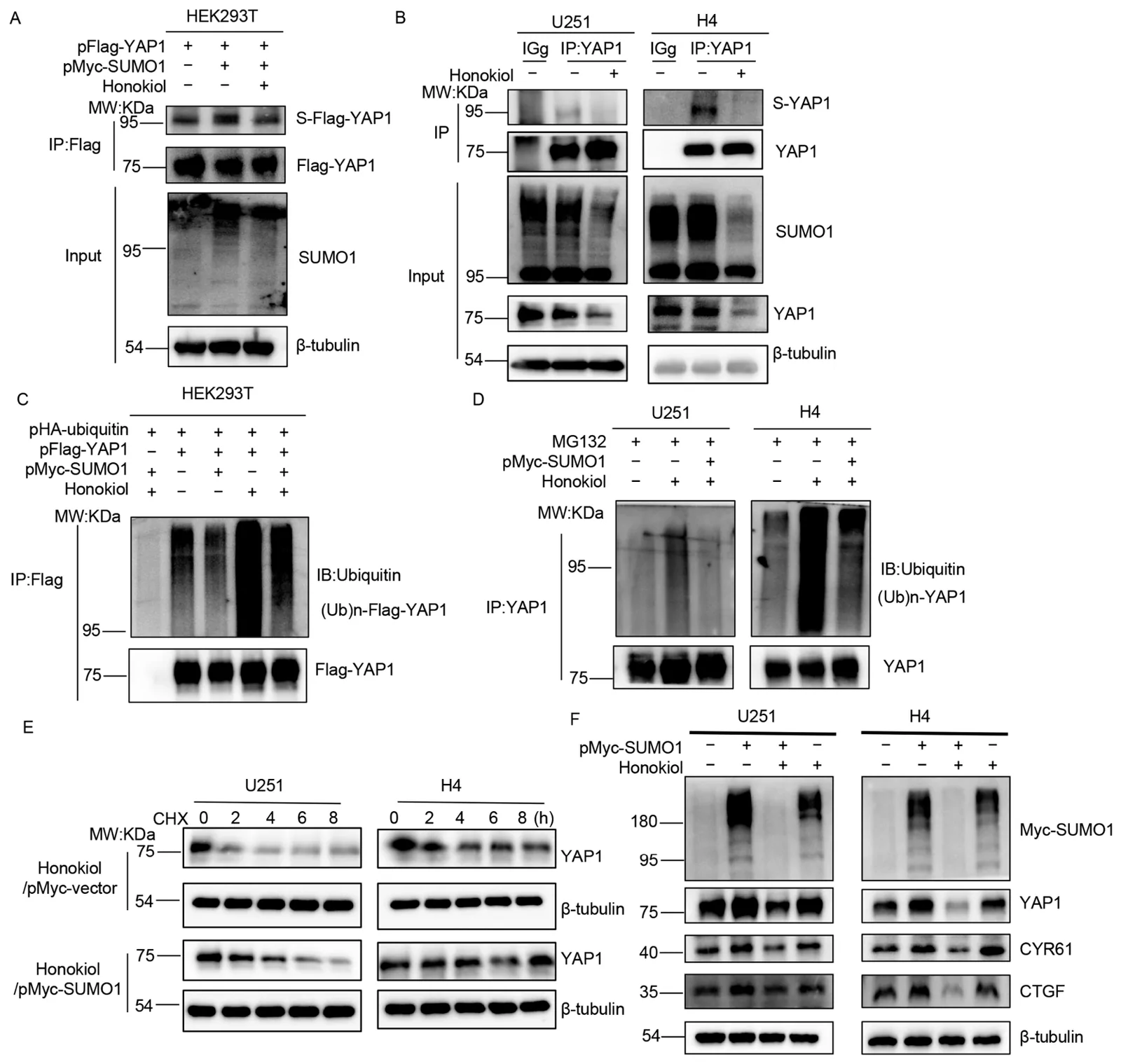
SUMO1 over-expression partially attenuates HNK-induced degradation of YAP1. (**A**) CO-IP assay displaying the effect of HNK on YAP1 SUMOylation. HEK293T cells were transiently co-transfected with pFlag-YAP1 and pMyc-SUMO1 plasmids for 24 h, then treated with or without 60 μM HNK for 24 h. Cell lysates were performed with IP to capture Flag-tagging YAP1, which was immunoblotted with FLAG and SUMO1 antibodies. (**B**) CO-IP assay displaying the effect of HNK on the SUMOylation of endogenous YAP1 in U251 and H4 cells. After treating with or without 60 μM HNK for 24 h, the cells were harvested. Cell lysates were performed with IP to capture YAP1, which was immunoblotted with YAP1 and SUMO1 antibodies. (**C**) CO-IP assay displaying the effect of SUMO1 on HNK-induced YAP1 ubiquitination. HEK293T cells were transiently co-transfected with pFlag-YAP1 and pHA-ubiquitin plasmids for 12 h, then transfected with pMyc-SUMO1 plasmids for 24 h. After treating with or without 60 μM HNK for 24 h, the cells were harvested. Cell lysates were performed with IP to capture Flag-tagging YAP1, which was immunoblotted with FLAG and ubiquitin antibodies. (**D**) CO-IP assay displaying the effect of SUMO1 on HNK-induced endogenous YAP1 ubiquitination. U251 and H4 cells were treated with or without 60 μM HNK for 24 h, then cells were treated with 20 μM MG132 for 8 h before harvest. Cell lysates were performed with IP to capture YAP1, which was immunoblotted with YAP1 and ubiquitin antibodies. (**E**) To evaluate the effect of SUMO on HNK-induced YAP1 instability, SUMO1-transfected U251 and H4 cells were treated with or without 60 µM HNK in the presence of 100 µM CHX for the indicated time kinetics. (**F**) Western blotting analysis of the YAP1, CTGF and CYR61 expression in SUMO1-transfected U251 and H4 cells treated without or with 60 μM HNK for 24 h. (Ub)n-Flag-YAP1: the ubiquitinylated Flag-tagging YAP1, (Ub)n-YAP1: the ubiquitinylated YAP1, S-Flag-YAP1: SUMOylated Flag-tagging YAP1, S-YAP1: SUMOylated YAP1, IP: immunoprecipitation, IB: immunoblot, Input: same account of cell lysate to load. The original Western blot images can be found in the Supplementary Materials.

Previous studies have indicated that SUMO1 enhances YAP1 protein stability by suppressing its ubiquitylation [28]. To validate the involvement of SUMOylation in HNK-induced YAP1 degradation, we analyzed YAP1 SUMOylation in response to HNK treatment. As shown in Figure 6A,B, HNK treatment inhibited YAP1 SUMOylation. Notably, SUMO1 over-expression markedly increased the abundance of SUMOylated YAP1 (Figure 6A, lane 2), and this increase was partially attenuated by HNK treatment (Figure 6A, lane 3), indicating that HNK partially counteracts the elevation of YAP1 SUMOylation induced by SUMO1 over-expression.

To further determine the role of SUMO1 in HNK-induced YAP1 ubiquitylation, we transiently transfected cells with the pMyc-SUMO1 plasmid for SUMO1 overexpression. As shown in Figure 6C,D, co-expression of SUMO1 reduced HNK-induced YAP1 polyubiquitylation. Furthermore, following HNK treatment, cells transfected with the pMyc-vector control exhibited more rapid YAP1 protein degradation than did SUMO1-overexpressing cells (Figure 6E). In addition, SUMO1 overexpression partially attenuated the HNK-mediated suppression of YAP1, CYR61 and CTGF protein expression (Figure 6F). Collectively, these findings indicate that HNK modulates YAP1 expression at the post-translational level, and that the HNK-induced promotion of YAP1 ubiquitylation and destabilization is partially counteracted by SUMO1 over-expression.

## 4. Discussion

Among primary central nervous system malignancies in adults, malignant glioma ranks first in incidence, with GBM standing as its most aggressive phenotype. GBM pathogenesis presents a formidable therapeutic challenge, as single-agent interventions targeting linear cascades have consistently proven inadequate [30,31]. This inherent complexity has necessitated a strategic pivot toward plurifunctional targets capable of simultaneously resetting multiple aberrant nodes. In this regard, as a PTM that governs the stability, localization, and function of thousands of substrate proteins involved in proliferation, apoptosis, and migration, SUMOylation constitutes a compelling nodal point for pharmacological intervention [9].

While several small-molecule inhibitors directed against SENPs, SAE1, and UBC9 have been developed, their translational potential is severely constrained by poor BBB permeability and, in many instances, insufficient suppression of global SUMOylation in the GBM microenvironment [32,33]. In contrast, HNK, a natural biphenolic compound extracted from the traditional Chinese medicine *Magnolia* species, not only exhibits favorable BBB penetrance in vitro and in vivo, but also demonstrates a favorable safety profile in normal neural cells, positioning it as a uniquely viable scaffold for GBM-directed therapy [11,34,35]. Previous studies have systematically demonstrated, using X-ray crystallography, ^1^H–^15^N heteronuclear single quantum correlation, NMR chemical shift perturbation experiments, and molecular dynamics simulations, that certain phenolic derivatives allosterically inhibit SUMOylation via ligand-induced rigidification of Ubc9, the sole SUMO E2 enzyme [27]. Building upon this evidence, we further investigated whether HNK, another phenolic derivative, could suppress SUMOylation levels in glioma cells. Our data indicate that HNK treatment results in a more pronounced suppression of SUMO1 conjugation compared with that of SUMO2/3, suggesting a differential regulatory effect on SUMO paralogs without appreciable changes in the expression of core SUMOylation enzymes. Although the proposed allosteric rigidification mechanism requires further structural validation through techniques such as surface plasmon resonance, isothermal titration calorimetry, or X-ray crystallography, our findings are consistent with HNK functioning as a novel SUMOylation inhibitor. Moreover, the incomplete rescue of the effects of HNK against GBM by SUMO1 overexpression intriguingly suggests that HNK’s biological activity may partially transcend its direct impact on the SUMOylation cascade, hinting at broader substrate-specific regulatory interactions.

Given the pleiotropic nature of SUMOylation [36], we employed unbiased label-free proteomic profiling to delineate the global landscape of HNK action. Among the approximately 200 proteins exhibiting over 2-fold accumulation following HNK treatment, YAP1, a principal downstream effector of the Hippo pathway governing cell proliferation, growth, and apoptosis, emerged as a particularly salient candidate [37,38,39,40,41,42,43,44,45,46]. The oncogenic relevance of YAP1 in GBM is well-documented; its amplification, along with that of its target genes CYR61, CTGF, and BIRC5, is significantly enriched in GBM specimens, where elevated YAP1 activity drives proliferative vigor and confers resistance to radiotherapy [43,44,45]. Moreover, recent evidence implicates YAP1 in promoting GBM cell migration and invasion through transcriptional regulation of N-cadherin and Twist [46]. Collectively, these observations indicate that YAP1 represents a priority target in GBM, although its therapeutic modulation remains challenging. Our study further reveals that HNK primarily acts on the Hippo pathway through direct suppression of YAP1, rather than via LATS1-mediated regulation of YAP1 upstream. Moreover, our research shows that HNK orchestrates YAP1 downregulation at the PTM level. Through the use of the proteasome inhibitor MG132 and targeted ubiquitination assays, we indicate that HNK actively promotes polyubiquitination of YAP1, subsequently routing it for proteasomal degradation, which is a mechanism consistent with emerging reports of HNK’s capacity to induce substrate degradation via the ubiquitin–proteasome system [47,48].

YAP1 was previously identified as a bona fide SUMO1 substrate, with SUMO1 conjugation protecting it from ubiquitylation-mediated degradation [28]. Both SUMOylation and ubiquitylation were found to target the same lysine residues, K97 and K242, implying a competitive regulatory mechanism at these sites [28]. In line with these findings, we observed that SUMO1 overexpression elevated YAP1 levels in two glioma cell lines, reinforcing the notion that SUMOylation is critical for YAP1 stability in this malignancy. Furthermore, forced SUMO1 overexpression partially abrogated the HNK-induced suppression of YAP1, CYR61, and CTGF, suggesting that deSUMOylation may serve as an initiating trigger for YAP1 destabilization. Nevertheless, we acknowledge that these rescue experiments, which rely on global SUMO1 overexpression, cannot definitively distinguish YAP1-specific effects from broader alterations across the SUMOylated proteome. Definitive validation of this mechanism will require future studies using SUMOylation-deficient YAP1 mutants to determine whether abrogation of YAP1 SUMOylation alone is sufficient and required for HNK-mediated destabilization. Despite this limitation, our findings collectively point to a novel regulatory cascade wherein HNK shifts the post-translational modification balance from SUMOylation-mediated stabilization toward ubiquitylation-driven degradation, thereby functionally inactivating the oncogenic YAP1 axis.

Moerover, given the broad impact of HNK on global SUMOylation and the observation that YAP1 overexpression only partially rescues HNK-induced growth inhibition in glioma cells, it is reasonable to postulate that HNK may exert its anti-proliferative effects through a multi-pathway mechanism beyond YAP1 alone. Specifically, HNK may function as a deSUMOylating agent that concurrently modulates several signaling cascades known to be both SUMO substrates and HNK targets, including the P53, NF-κB, and AKT pathways, as well as the cell cycle regulator CDK6 [10,49,50,51]. The collective perturbation of these pathways likely synergizes to induce comprehensive glioma cell growth arrest. Taken together, our results expanded a mere description of HNK’s bioactivity and propose a conceptual framework for a previously unrecognized therapeutic axis of SUMOylation-dependent regulation of YAP1 in glioma. By verifying that pharmacological interference with SUMOylation can effectively destabilize a major oncogenic driver, our work suggests a strong rationale for a paradigm-shifting inhibitor design strategy, namely targeting the SUMO–YAP1 interaction interface rather than the YAP1 protein itself. This indirect approach may circumvent the toxicity and resistance commonly associated with direct YAP1 inhibitors. Given HNK’s favorable BBB permeability and low intrinsic toxicity, our findings advocate for the continued optimization of HNK or its derivatives as a first-in-class therapeutic modality that addresses the network-level complexity of GBM, while also laying the groundwork for future structure–activity relationship studies aimed at enhancing its selectivity for the SUMO1–YAP1 axis.

## 5. Conclusions

**Figure 7:**
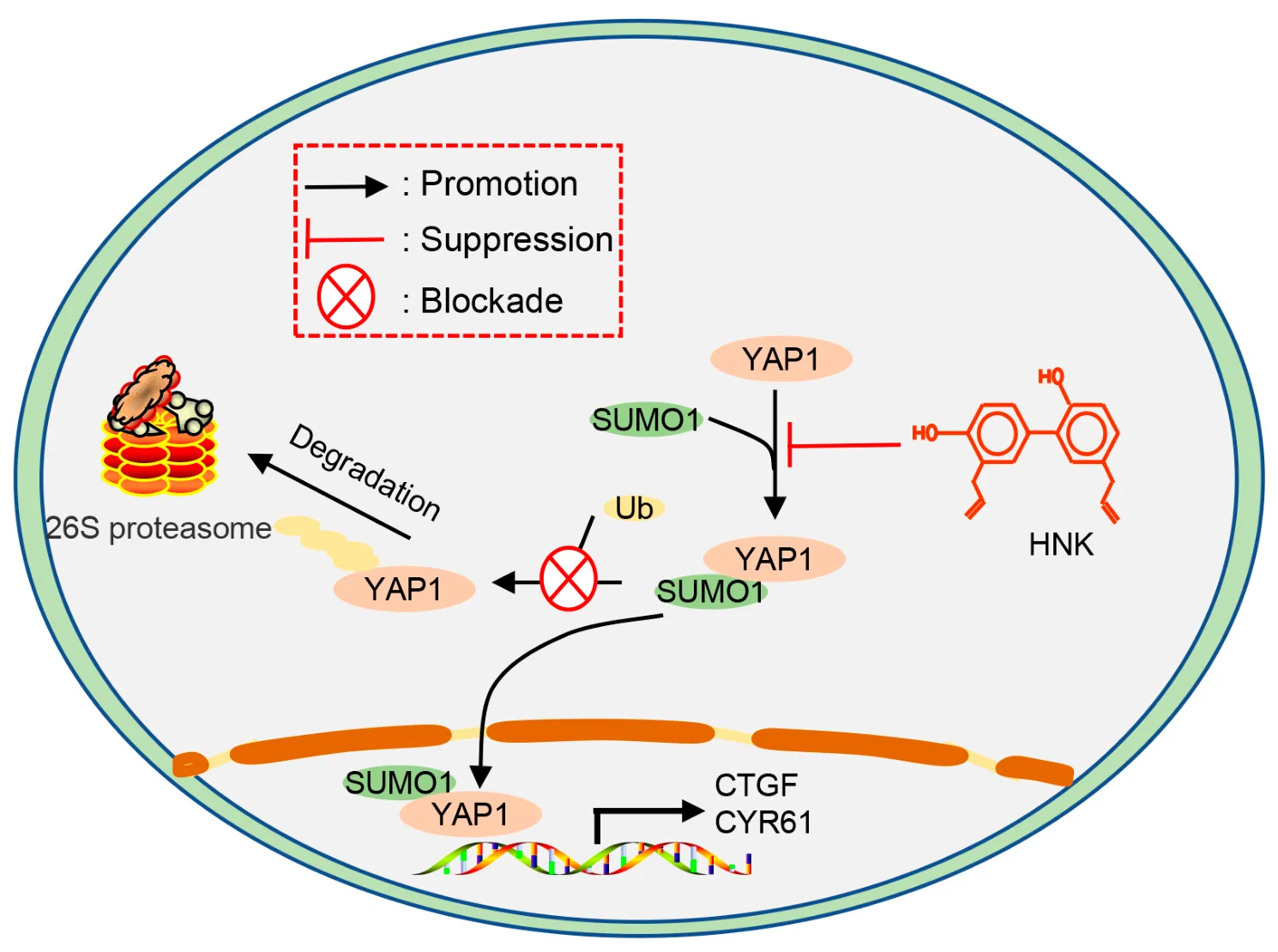
A working mechanism model of HNK-induced YAP1 ubiquitin–proteasome degradation by disrupting SUMO1-YAP1 conjugation, ultimately resulting in the inactivation of YAP1 signaling pathway and cell proliferation inhibition.

In this study, we identified HNK as a novel inhibitor of global SUMOylation and a potential adjuvant therapeutic agent for GBM. Label-free proteomic analysis revealed that HNK induced alterations in the abundance of multiple proteins, including YAP1. Mechanistically, HNK disrupted SUMO1-YAP1 conjugation and promoted YAP1 degradation via the ubiquitin–proteasome pathway, ultimately leading to the inactivation of YAP1 signaling and suppression of cell proliferation (Figure 7). Collectively, our findings establish HNK as a novel global SUMOylation inhibitor, providing a new lead compound for the development of SUMO-targeted inhibitors.

## Data Availability

The original contributions presented in the study are included in the article/Supplementary Materials, and further inquiries can be directed to the corresponding authors.

## Supplementary Materials

The following supporting information can be downloaded at https://www.mdpi.com/article/10.3390/biom16081158/s1, Table S1: Total protein differences identified by LC-MS/MS (U251-HNK versus U251-DMSO); Table S2: KEGG pathway analysis of HNK-induced differential expression proteins in U251 cells; The original Western blot images can be found in the Supplementary Materials.
